# Association of Type of Vaccination Center With Time to Emergency Department Presentation for Acute COVID-19 Infection: An Exploratory Analysis

**DOI:** 10.7759/cureus.51229

**Published:** 2023-12-28

**Authors:** Timothy Regan, Walter B Wills, Andrew R Barbera, Pedro E Reyes, Kellcee Jacklin, Dana Crowder, Kathryn Henderson, Brandon Montes, Andrew Bugajski

**Affiliations:** 1 Department of Emergency Medicine, Lakeland Regional Health, Lakeland, USA; 2 Department of Research and Sponsored Studies, Lakeland Regional Health, Lakeland, USA

**Keywords:** post vaccine, vaccine efficacy, vaccine logistics, vaccine science and policy, covid-19 vaccine

## Abstract

Objective

The objective of this study was to identify potential associations between coronavirus disease 2019 (COVID-19) vaccination center reception location and time to presentation to the emergency department for acute COVID-19 infection. The a priori hypothesis was that there are significant differences in the outcome based on vaccination administration center type.

Methods

This was a cross-sectional, observational study conducted within a hospital in Lakeland, Florida, between October 2021 and May 2022. Participants were at least 18 years old with confirmed severe acute respiratory syndrome coronavirus 2 (SARS‑CoV‑2) infection and at least two COVID-19 symptoms at enrollment. Patients with prior confirmed COVID-19 diagnosis and hospitalization within 10 days of screening were excluded. Participants were sampled from within the emergency department of the institution. The primary outcome was time to presentation to the emergency department for acute COVID-19 infection since the last vaccination dose from each sampled COVID-19 vaccination center location.

Results

A total of 93 participants were analyzed. Of these, 48 (52%) participants received COVID-19 vaccination. Participants vaccinated at vaccine clinics demonstrated a significantly longer mean survival time (288.2 (29.9)) compared to other sites. Significant predictors of hospitalization were age (aOR, 1.09, 95%CI 1.02-1.16, p < 0.01), sex (aOR: 10.05, 95%CI 1.52-66.54, p < 0.05), physical function (aOR, 0.90, 95%CI 0.83-0.97, p < 0.01) and number of medications (aOR, 1.34, 95%CI 1.14-1.58, p < 0.001).

Conclusions

This exploratory analysis highlights the need for further investigation into both characteristics of healthcare institutions and individual-level factors that may play a role in the prolonged prevention of emergency department presentations due to COVID-19 infection. Increased transparency of data regarding practices related to the administration of COVID-19 vaccines across various institutions may be beneficial in further understanding the role of COVID-19 vaccinations in preventing symptomatic disease across local and global communities.

## Introduction

The coronavirus disease 2019 (COVID-19) pandemic invoked a need for the rapid development and dissemination of new COVID-19 vaccines, which were first administered to the general public in December 2020 [1]. Vaccination was an utmost public and governmental priority, for which governments were tasked with sustaining a robust vaccination distribution system for a high volume of the global population [2-4]. The newly developed mRNA vaccines developed by Pfizer and Moderna showed the most promise to mitigate morbidity and mortality from COVID-19 [5,6]. However, the emergence of this new technology exacerbated existing societal barriers to vaccine uptake (general vaccine hesitancy, psychological distress, geographic isolation [7-9]) and added new logistic obstacles specific to the mRNA vaccines.

One major logistic obstacle was the distribution and storage of the mRNA vaccines, which needed to be held at temperatures as low as -90°F to protect the vaccine from physical and chemical degradation [10]. The rapidity of this national distribution posed a new barrier, in that most institutions did not readily have access to dedicated freezers to store the vaccines [11]. In addition to strict storage requirements, there were strict preparation and administration requirements that left each vial of vaccine only appropriate for use for up to 12 hours post-thaw [12]. Despite these challenges, COVID-19 vaccinations were being distributed nationwide via five main modalities: vaccination clinics, hospitals, retailers, pharmacies, and urgent care centers, where up to 4.5 million doses per day were administered at peak volume by April 2021 [13]. Given the rapidity of these efforts, the logistic intricacies, and the existing societal barriers to vaccine uptake, there is no readily available data to infer associations that may approximate the potential ill effects of variations in institutional procedures (based on institution type) related to the distribution, storage, and administration of these mRNA vaccines.

Accordingly, the purpose of this study was to explore and contextualize a cohort of vaccinated and unvaccinated patients who presented with acute COVID-19 infection and explore which factors may have played a role in those patients seeking care. The primary aim of this study was to explore associations between COVID-19 vaccination administration center location (e.g., primary/urgent care, vaccine clinics, hospitals, retailers, pharmacies) and time to presentation to the Emergency Department (ED) for acute, symptomatic COVID-19 infection. The secondary aim of this study was to identify potentially relevant factors associated with hospitalization for primary COVID-19 infection, such as COVID-19 vaccination status, geography (distance from the ED), anxiety or depressive symptoms, and physical functioning while controlling for known contextual variables (age, sex, body mass index [BMI]), comorbidities, medication history, and inflammatory biomarkers [14]). Taken together, these aims seek to explore potential differences in time to ED presentation for symptomatic infection of COVID-19 after receiving vaccination as well as to explore predictors of severe infections requiring hospitalization across the entire sample.

## Materials and methods

Study design and setting

This study was a cross-sectional, observational study design, conducted at an 864-bed regional hospital in Lakeland, Florida, United States, from October 2021 to May 2022. The study was approved by the Institutional Review Board of Lakeland Regional Health Systems Inc. (IRB#000021260, ethical approval number: 1800399). Prior to enrollment, participants were required to sign an informed, written consent for their participation in the study. All data were collected via patients’ self-reported surveys at the time of hospitalization, and supplemented by manual electronic medical record (EMR) review. This study followed the Strengthening the Reporting of Observational Studies in Epidemiology (STROBE) guidelines for the reporting of cross-sectional studies [15].

Patient population

Patients were sampled via non-probability sampling. Patients were approached in the ED once they were coded as seeking treatment for a primary COVID-19 infection and ED physician notes within the EMR identified the patient to be admitted as an inpatient or discharged from the ED. Non-probability sampling refers to a non-random sampling method by which participants are sampled based on their accessibility to the research team. Participants were identified for potential enrollment twice daily into the study using an EMR query of COVID-19 test results for patients currently admitted to the ED. To enroll in the study, individuals were included if they were 18 years old or greater, had a confirmed severe acute respiratory syndrome coronavirus 2 (SARS‑CoV‑2) infection by any authorized or approved polymerase chain reaction (PCR) or antigen test collected within 10 days of screening, and had two or more current symptoms of acute COVID-19 infection for less than or equal to seven days. Participants were excluded from the study if they had a prior diagnosis of COVID-19 infection greater than 10 days prior to screening, or current or recent hospitalization (within 10 days of screening) for any reason.

Measurements

An investigator-designed vaccination profile questionnaire was administered to collect data regarding each participant’s COVID-19 vaccine history (e.g., if they had received vaccination (yes/no), what brand was received (e.g., Moderna, Pfizer, Johnson & Johnson), number of doses received, administration of a booster dose, and associated dates of each dose received). Additionally, the type of COVID-19 vaccination administration center used for the analysis of the primary outcome of the present study was sampled using this questionnaire obtained via self-report from participants (e.g., vaccine clinic, pharmacy, retailer, urgent/primary care, hospital, other location). To ensure the accuracy of this data, members of the research team used a combination of techniques (triangulation) to validate responses including family member reconciliation, cross-referencing in-network records, and contacting external providers [16]. The data obtained from this questionnaire was utilized to characterize the vaccination status of the sample in descriptive analyses.

Anxiety and depressive symptoms were assessed to address the unique situation of the COVID-19 pandemic, in which there was societal insecurity with the virus and emerging technologies. To capture acute anxiety and depression, the Hospital Anxiety and Depression Scale (HADS) was used [17]. The HADS is composed of two seven-item subscales, HADS-Anxiety and HADS-Depression, which assess clinically relevant symptoms of each illness. The HADS subscales were utilized to assess the impact of anxiety or depressive symptoms in contributing to the likelihood of hospitalization as a result of COVID-19 infection.

Physical function was assessed utilizing the Patient-Reported Outcomes Measurement Information System® (PROMIS®) Short Form V2.0, Physical Function 6b, a six-item instrument assessing current limitations on physical function [18]. This was included to contextualize and infer the effect in which physical impediment may play a role in a patient’s decision to seek care in the study institution’s rural catchment area.

Prespecified clinical covariates were manually extracted from the participant’s EMR, which included: age (in years), biological sex, BMI, distance from the ED (calculated from reported address of residence to ED, in miles), total number of documented comorbidities, medications (number of, dose, and schedule per medication), and biomarkers of inflammation (e.g., C-reactive protein (CRP), troponin, lactate, and D-dimer).

Outcome

The primary outcome variable was defined as the time in days calculated from the last reported dose of COVID-19 vaccination to the date of presentation to the ED. This measure was used as the primary endpoint for our study when assessing between-group differences based on COVID-19 vaccination distribution institution modalities. 

Statistical analysis

Data analyses were performed using IBM SPSS Statistics for Windows, Version 28.0 (Released 2021; IBM Corp., Armonk, New York, United States) [19]. Additionally, an a priori sample size analysis was conducted demonstrating approximately 108 cases would be required to detect a large effect size δ = 0.7 with 80% power utilizing the Log-Rank test; all analyses were specified priori α = 0.05 [20]. Descriptive statistics (e.g., means, standard deviations, frequencies, and proportions) were analyzed first to explore the degree of data integrity and missingness, as well as to characterize the sample within the confines of the included variables. Multiple imputation techniques were subsequently employed among specified variables. Bivariate hypothesis testing occurred evaluating two-sided alternative hypotheses and was conducted utilizing independent samples, t-testing for normally distributed continuous variables, and Chi-Square analyses for categorical variables. The Mann-Whitney U test was employed when continuous variables violated the assumption of normality. Normality was assessed for each variable using values of kurtosis and skew and visual inspection of the resulting histogram. Only those participants with complete data on the investigator-designed Vaccination Profile Questionnaire were included in the analytic sample; this approach was used to account for interval-censored data as it related to the validity of the time-to-event analyses [21]. Kaplan-Meier estimates and log-rank tests were used to examine potential differences in time-to-presentation to the ED due to COVID-19 infection in relation to the various types of vaccination distribution centers (primary/urgent care, vaccine clinics, hospitals, retailers, and pharmacies). Patients across each vaccine distribution center were modeled as a function of time to approximate the time to ED presentation for COVID-19 infection by each center.

To examine the secondary aim of the study, a multivariable hierarchical logistic regression model was constructed to investigate the potential predictors of hospitalization due to COVID-19 infection. Variables were input hierarchically based on their relationship with the response variable demonstrated in bivariate hypothesis testing and if theorized to significantly impact the outcome from prior literature. Variables were retained in the final model based on improvement in overall model fit demonstrated with their addition, as indicated by a significant reduction in the -2 Log-Likelihood statistic. Assumptions of logistic regression were met prior to interpreting the results (linearity of logit, independence of errors, normality, equality of variances, and lack of multicollinearity [22]).

## Results

Demographic characteristics of participants

An analytic sample of 93 cases was included in this analysis. There were 48 (52%) participants who reported being vaccinated in the sample, all of whom received either Moderna or Pfizer COVID-19 vaccinations. Among the vaccinated subsample with the study, the most vaccinations were administered at pharmacies and hospitals (n=14, 29% each) and the fewest (n=2, 4%) were given at locations other than the five primary vaccination distribution modalities assessed by the investigator-designed vaccine profile questionnaire (urgent/primary care centers, hospitals, pharmacies, retailers, vaccine clinics). The entire vaccinated subsample reported being fully vaccinated at the time of data collection (having received two doses); however, only 10 (21%) participants who were vaccinated reported having previously received a booster dose, regardless of vaccination brand. The mean (standard deviation) age of the sample was 52.7 (17.0) years, and the average BMI was 35.8 (12.5) kg/m^2^. The sample demonstrated an average HADS-Anxiety and median (interquartile range) HADS-Depression score below the threshold for clinically relevant symptoms of either disorder (5.7 (4.3) and 3 (1-4), respectively). There were 71 (76%) participants who were discharged while 22 (24%) were admitted. The complete sample characteristics may be viewed in Table 1.

**Table 1:** Sample characteristics by discharge disposition For normally distributed continuous variables, independent samples t-testing was utilized for bivariate hypothesis testing. For non-parametric continuous variables, Mann-Whitney U testing was utilized. For bivariate hypothesis testing with categorical data, Chi-Square analysis was utilized. For categorical data violating the assumptions of Chi-Square analyses, Fisher’s exact test was utilized. Effect size reported as Cohen’s d for continuous, parametric data, effect size estimate r for non-parametric continuous data, Phi φ for 2 x 2 contingency tables, and Cramer’s V for greater than 2 x 2 contingency tables. ^a^p < .05, ^b^p < .01, ^c^p <.001.^d^Proportions represent a subsample including only those who reported having received COVID-19 vaccination HADS: Hospital Anxiety-Depression Scale; IQR: Interquartile Range; PROMIS®: Patient-Reported Outcomes Measurement Information System®

Variable	Total (n = 93)	Admitted (n = 22)	Discharged (n = 71)	Effect Size
Sex (Female), n (%)	50 (54%)	7 (32%)	43 (61%)	.293^a^
Age (years), mean (SD), years	52.65 (16.95)	61.18 (19.19)	50.00 (15.40)	-.684^b^
BMI (kg/m^2^), mean (SD)	35.76 (12.46)	36.03 (13.52)	35.68 (12.21)	.450
Distance to ED (miles), median (IQR)	10 (5.75-14.50)	8.80 (4.83-13.83)	10 (6-15)	-.059
Number of comorbidities, median (IQR)	4 (1-5)	5 (2.75-7.25)	3 (1-5)	.291^b^
Number of medications, median (IQR)	5 (1-10)	13 (5.75-18.25)	4 (1-7)	.440^c^
Vaccinated, n (%)	48 (52%)	9 (41%)	39 (55%)	-.119
Vaccination brand^d^				.109
Moderna	30 (63%)	5 (56%)	25 (64%)	
Pfizer	17 (35%)	4 (44%)	13 (33%)	
Moderna & Pfizer	1 (2%)	0 (0%)	1 (3%)	
Vaccine Location^d^, n (%)				.372
Pharmacy	14 (29%)	4 (44%)	10 (26%)	
Hospital	14 (29%)	1 (11%)	13 (33%)	
Vaccine clinic	7 (15%)	3 (33%)	4 (10%)	
Urgent/primary care	5 (13%)	0 (0%)	6 (15%)	
Retailer	5 (10%)	1 (11%)	4 (10%)	
Other	2 (4%)	0 (0%)	2 (5%)	
Received booster^d^, n (%)	10 (21%)	2 (22%)	8 (20%)	.016
Time to seeking care^d^ (days), mean (SD)	181.54 (92.82)	236.11 (122.55)	168.95 (84.40)	-.747
HADS-Anxiety score, mean (SD)	5.67 (4.32)	12.09 (7.26)	5.92 (4.46)	.243
HADS-Depression score, median (IQR)	3 (1-4)	2.50 (1-7.50)	3 (1-4)	.032
PROMIS® Physical Function score, median (IQR)	46.10 (36.45-59.00)	37.3 (26.45-50.90)	46.20 (39.50-58.80)	-.255
Vaccine Hesitancy Confidence score, median (IQR)	5 (3.33-6)	4.17 (2.92-6.08)	5 (3.67-6)	-.076
Vaccine Hesitancy Complacency score, median (IQR)	3 (1.33-3.67)	3.17 (1.58-4)	3 (1.33-3.67)	.105
Vaccine Hesitancy Calculation score, median (IQR)	6.67 (5-7)	7 (4.83-7)	6.33 (5-7)	.032
Vaccine Hesitancy Collective Responsibility score, median (IQR)	4.67 (4-5)	4.83 (4-5)	4.67 (4-5)	-.011
Vaccine Hesitancy Constraints score, median (IQR)	1 (1-2.33)	1.17 (1-3)	1 (1-2)	.082

Independent samples t-testing indicated there were significant differences in age observed between those admitted and discharged in the sample (t = -2.98. p = 0.003, d = -0.684). Non-parametric Mann-Whitney U analyses and Fisher’s exact test demonstrated significant differences in the number of comorbidities (U = 1088.50, p = 0.005, r = 0.291) number of medications (U = 1247.00, p < 0.001, r = 0.440), and proportion of females (p = 0.02, φ = 0.293) between those admitted and discharged in the sample. There were no significant differences in discharge disposition based on vaccination status. The complete results of bivariate hypothesis testing may be viewed in Table 1.

Inferential analyses

**Figure 1:**
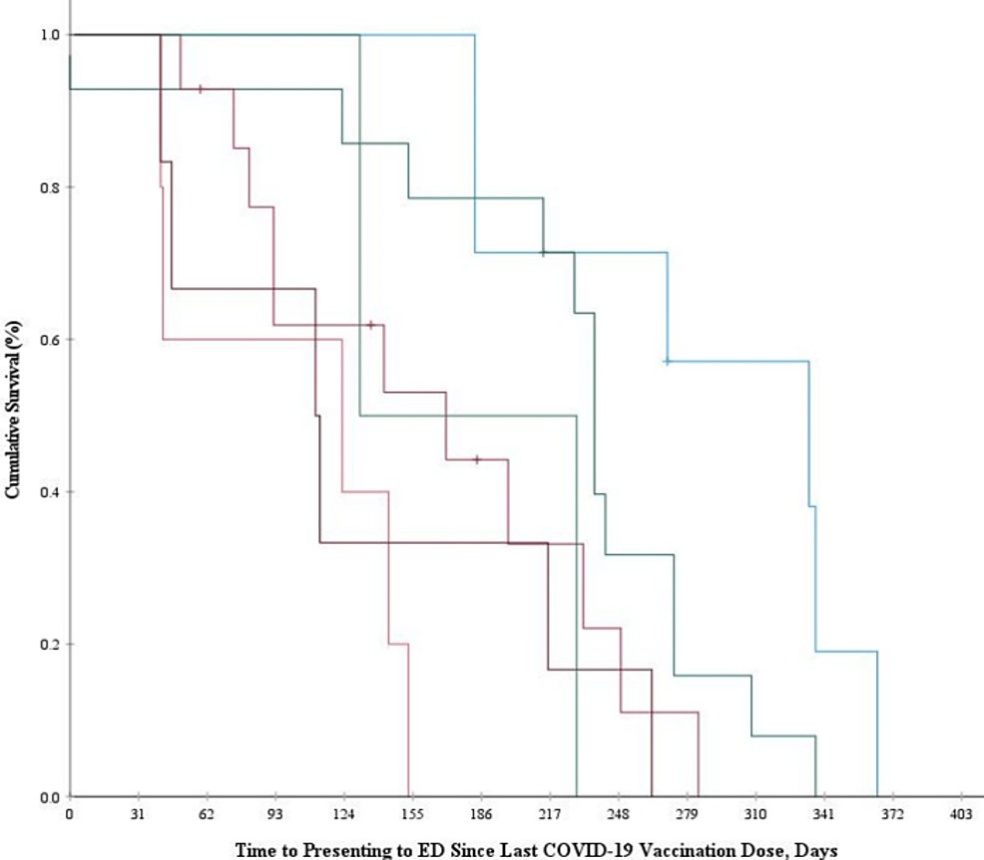
Kaplan-Meier survival curves for time-to-presenting to emergency department since last COVID-vaccination dose by vaccination location X Axis: Time to presenting to ED since last COVID-19 vaccination dose (Days); Y Axis: cumulative survival (%) Color Legend: Light Blue (Vaccine Clinic), Dark Green (Hospital), Magenta (Pharmacy), Orange (Retailer), Maroon (Primary Care), Light Green (Other Locations) COVID-19: coronavirus disease 2019

Kaplan-Meier survival analysis was conducted to examine the associations of vaccination administration modality and time to seeking care in the emergency department as measured from the last dose of COVID-19 vaccination (Day 0). The longest mean survival time was 288.2 (29.9) days for those who received their vaccine at a vaccine clinic, and the shortest mean survival time was 100.6 (24.6) days for those who received vaccination at a retailer. There were significant overall differences in time to seeking care among the sample when stratified by vaccination distribution location, Log-rank test χ2(5) = 23.01, p <0.001. Significant differences were observed between vaccine clinics and all distinct locations, with the exception of hospitals (χ2 = 2.84, p = 0.09). A visual representation of this analysis is given in Figure 1.

Multivariate predictors of hospitalization

When examining the secondary objective of the present study, the multivariable hierarchical logistic regression model suggested that increased age (β = 0.09, OR 1.09, 95%CI 1.02-1.16, p < 0.01), increased number of medications (β = 0.29, OR 1.34, 95%CI 1.14-1.58, p < 0.001), male sex (β = 2.31, OR 10.05, 95%CI 1.52-66.54, p < 0.05), and reduced physical function (β = -1.09, OR 0.90, 95%CI 0.83-0.97, p < 0.01) were significant predictors of hospitalization for primary COVID-19 infection, while controlling for BMI, comorbidities, distance to the ED, and vaccination status. These predictors were included in the final model due to a significant reduction in the -2 Log-Likelihood statistic occurring upon their addition during the hierarchical model-building process. This model explained 47% of the variance in scores related to hospitalization in the sample (Table 2).

**Table 2:** Unadjusted and adjusted logistic regression models for hospitalization The adjusted model included: age in years, vaccination status, number of comorbidities, number of medications, PROMIS Physical Function scores, biological sex, BMI, and distance to ED. R^2^ = 0.47 (Hosmer & Lemeshow), 0.398 (Cox & Snell), 0.605 (Nagelkerke). Model χ^2^(8) = 46.740, p < 0.001. For Odds Ratios: ^a^p < 0.05, ^b^p < 0.01, ^c^p < 0.001. aOR: Adjusted Odds Ratio; PROMIS®: Patient-Reported Outcomes Measurement Information System®

Variable	Unadjusted Model, OR (95% CI)	Adjusted Model, aOR (95%CI)
Age, years	1.05 (1.01 – 1.08)^b^	1.09 (1.02 – 1.16)^b^
Vaccination Status		
Vaccinated	0.57 (0.22 – 1.50)	0.28 (0.06 – 1.39)
Not vaccinated	1 (Reference)	1 (Reference)
Number of comorbidities	1.06 (0.97 – 1.16)	0.95 (0.78 – 1.16)
Number of medications	1.21 (1.10 – 1.32)^c^	1.34 (1.14 – 1.58)^c^
PROMIS® Physical Function score	0.94 (0.90 – 0.98)^b^	0.90 (0.83 – 0.97)^b^
Sex		
Female	1 (Reference)	1 (Reference)
Male	3.29 (1.19 – 9.09)^a^	10.05 (1.52, 66.54)^a^
BMI (kg/m^2^)	1.00 (0.96 – 1.04)	0.98 (0.93 – 1.04)
Distance to ED (miles)	0.98 (0.92 – 1.04)	0.97 (0.89 – 1.06)

## Discussion

In this study, we demonstrate novel evidence suggesting that there may be significant differences in time to seeking care for acute COVID-19 infection based on the reception of COVID-19 vaccine doses in the various vaccination administration centers sampled, as those who received vaccination at vaccine clinics took significantly longer (in days) to present to the ED for treatment due to confirmed, symptomatic COVID-19 infection when compared to all other distinct locations (with exception to hospitals) after their most recent COVID-19 vaccine dose. Additionally, in this study we examined relevant predictors of hospitalization for primary COVID-19 infection in corroboration with prior evidence. Whether this phenomena was observed due to differences in human behavior outside the scope of this investigation or differences in the storage, handling, and administration practices of the vaccination centers sampled is unclear. However, further discussion integrating our findings with available prior literature is warranted to best interpret these results for future investigation.

The present study demonstrated novel findings in suggesting individuals within the sample who received vaccination at a vaccine clinic took significantly longer to present to the ED for symptoms of primary COVID-19 infection when compared to other distinct locations, with the exception of hospitals (e.g., primary/urgent care centers, retailers, pharmacies). These findings may potentially be related to the storage, handling, and administration of these mRNA-lipid nanoparticle vaccines that required the aforementioned special freezing temperatures to prevent physical or chemical degradation. Although public data is sparse, there have been prior evidence elucidating the management of vaccinations in which the locations performing worse in our sample (e.g., primary/urgent care centers, retailers, pharmacies) have been subpar historically [23], and this problem may be exacerbated within low-middle income countries [24]. For example, Yuan et al. demonstrated that out of 138 general primary care offices, only 6% of practice staff answered all knowledge-based questions in assessments related to the storage and handling of vaccinations correctly, and only 11 vaccine-containing refrigerators had thermometers present [25]. Bell et al. also demonstrated that primary care centers which were compliant with vaccination refrigeration requirements were likely to be associated with hospitals and assigned their highest trained staff to the task of properly storing the vaccines [26]. In the present sample, hospitals and vaccine clinics performed similarly in prolonging the period of time between a vaccine dose and presentation to the ED. While there may be potentially other rationales for this phenomena, this evidence may suggest the persistence of relevant differences in the training of clinical staff in the administration and handling of these vaccinations compared to the other locations in our sample. These vaccine clinics were often established by the Federal Emergency Management Agency (FEMA) and were provided federal funding that supported the hiring of trained clinical personnel to supervise the administration of doses, as well as tangible property which may have included the resources to properly store the vaccine vials [27]. The provision of adequate resources and hyperspecialized staffing to the task of storing, handling, and administering vaccinations within these sites may explain why vaccine clinics performed similarly to hospitals in this regard. However, it should be recognized that individual-level behavioral factors were not investigated within the present study, and represent targets for future investigation prior to concluding that the phenomenon is solely a consequence of center-based differences. This was not able to be fully examined due to the current paucity of public data regarding these potential variations.

Additionally, the present study found that increased age, increased number of concomitant medications, reduced physical function and male sex were predictive of hospitalization for primary COVID-19 while controlling for demographic and clinical covariates. Our findings echo much of the current literature demonstrating that increased age may predict being hospitalized for COVID-19 [28-31] As individuals age, there may be multifaceted explanations of how COVID-19 may become more severe. Increased age may also help explain why greater amounts of concomitant medications being taken by patients may also have significantly predicted hospitalization, indicating a higher severity of comorbid disease that may have compounding effects on the symptoms and severity of a COVID-19 infection [29]. Interestingly, being vaccinated against COVID-19 did not show a significant reduction in the likelihood of experiencing hospitalization due to primary COVID-19 infection; however, this may be postulated to be due to the differences in the comorbidity burden and polypharmacy between these two groups at the time of data collection, as demonstrated by prior bivariate hypothesis testing. Although males were found to be at higher odds, it is against our better judgement given the wide confidence interval of the point estimate to generalize that this finding may be true in the population at large.

Limitations

The limitations of the present study must be acknowledged. First, the study was cross-sectional so causality may not be appropriately inferred. Second, due to temporal trends in COVID-19 incidence within our region, recruitment for the sample was closed prior to reaching the anticipated sample size; therefore, nonparametric analyses were employed to address our research question. Additionally, the investigation may not have been statistically powered to detect a difference among hospitalizations between our vaccinated and unvaccinated subsamples. Our recruitment period also encapsulated time periods encompassing both the Delta and Omicron variants of COVID-19, which may affect patients variably and alter results [32]. Lastly, as manual chart review data was dependent only on data entered into the EMR or ordered for the patient, many manual data collection points had missing data or were unusable in the final analysis (e.g., D-dimer, troponin, C-reactive protein, lactate).

## Conclusions

Our findings highlight the urgent need for a systemic review of vaccine storage and management procedures across different types of vaccination distribution and administration centers. This exploratory study also signals the need for greater transparency and availability of public data regarding the storage and handling practices of these vaccinations within the sampled locations. However, it should be noted that there are many potential individual and community-level factors that may also have impacted this phenomenon that were outside the scope of the present study. Yet, with the growing trend in mRNA vaccine technologies that will require similar storage and administration requirements to that of COVID-19 vaccines, our results likely impact future public health discussions and represent a call for the increased transparency of data regarding the management of vaccinations during public health crises across multiple institutions. Increases in available data regarding these concepts may allow future investigators to conduct detailed analyses of vaccination distribution patterns and procedures that may improve public health initiatives during times of crisis.
